# Unlocking Athletic Potential: The Integration of Chiropractic Care into the Sports Industry and Its Impact on the Performance and Health of Athletes and Economic Growth in China and Hong Kong

**DOI:** 10.7759/cureus.37157

**Published:** 2023-04-05

**Authors:** Andy Fu Chieh Lin, Shun Zhe Piong, William MH Wan, Peng Li, Valerie K Chu, Eric Chun-Pu Chu

**Affiliations:** 1 Chiropractic and Physiotherapy Centre, New York Medical Group, Hong Kong, HKG; 2 Sports Medicine, Sports Chiropractic Council of Hong Kong China, Hong Kong, HKG; 3 Global Initiatives China, Life University, Beijing, CHN; 4 Sports Medicine, Chongqing Technology and Business University, Chongqing, CHN; 5 Executive Board, Chiropractic Doctors Association of Hong Kong, Hong Kong, HKG

**Keywords:** chiropractic treatment, olympic, hong kong, sports medicine, chiropractic

## Abstract

This study explores the significant role chiropractic care can play in the sports industry, with a focus on countries like China and Hong Kong. As a vital component of sports medicine, chiropractic care can enhance athletic performance, health, and competitiveness by addressing biomechanical imbalances and optimizing neuromuscular function. The potential impact of chiropractic care on the sports industry includes attracting international events and investments, leading to increased economic opportunities, and the overall growth of the industry. Key strategies for promoting chiropractic care in the sports industry encompass developing a robust chiropractic care infrastructure, raising public awareness through targeted campaigns, and implementing supportive policies by governments and sports organizations. The integration of chiropractic care within sports medicine not only benefits individual athletes but also contributes to the broader development and success of the sports industry as a whole.

## Introduction and background

By focusing on the neuromusculoskeletal system and its optimization for athletic performance, sports chiropractic care has emerged as a critical component of the healthcare management of athletes [1]. It is a specialized approach that combines the principles of chiropractic care with an understanding of the unique demands placed on the bodies of athletes, thereby facilitating injury prevention, rehabilitation, and overall performance enhancement [2]. Chiropractic care includes a variety of manual therapies, such as spinal manipulation, mobilization, and soft tissue techniques, that target the musculoskeletal system to promote optimal function and relieve pain [3]. Several studies have found chiropractic care to be effective in treating common conditions seen in athletes, such as lower back pain, muscle strains, and joint dysfunctions [4,5]. In addition, the incorporation of chiropractic care into an athlete's healthcare regimen has been associated with enhanced biomechanics, decreased injury rates, and accelerated recovery [6].

In recent years, China and Hong Kong's sports industries have flourished due to increased investments in infrastructure, athlete development programs, and the hosting of world-class sporting events [7]. In contrast to more established markets such as the United States and Australia, however, the integration of chiropractic care into the landscape of sports medicine remains relatively limited. The Sports Chiropractic Council of Hong Kong, China, was founded in 2005 [8] to promote the role of chiropractic care in sports medicine and advocate for its incorporation into athlete healthcare management. This organization has played a significant role in promoting awareness and collaboration between healthcare professionals, athletes, and sports organizations. The objectives of the current study are to present a comprehensive review of the benefits of chiropractic care for athletes; provide an overview of the sports industry in China and Hong Kong, including the history of the Chiropractic Sports Council of Hong Kong, China; and discuss the potential impact of integrating chiropractic care into the sports industry, including policy recommendations and adoption strategies.

## Review

The role of chiropractic care in athlete performance

The role of chiropractic care in athlete performance derives from its fundamental principles and techniques, which focus on the assessment, diagnosis, and treatment of neuromusculoskeletal disorders and their effect on an individual's overall function [9,10]. Chiropractic care is founded on the premise that optimal nervous system function and musculoskeletal health depend on the proper alignment of the spine and other joint structures [11]. Chiropractors use various techniques and approaches, such as spinal manipulation, mobilization, soft tissue therapies, and rehabilitative exercises, to address biomechanical imbalances and improve an athlete's performance [2,12]. In the context of sports, common conditions treated by chiropractors include, but are not limited to, lower back pain, muscle strains, joint sprains, tendinopathies, and neural entrapments [4]. By addressing these issues with targeted chiropractic interventions, athletes can experience functional enhancements, decreased pain, and ultimately improved performance with high satisfaction [13].

Importance of chiropractic care for athletes

Chiropractic professionals treat patients with concussion-like symptoms, such as neck discomfort, headache, and dizziness and they also have exposure to athletes and teams in collision sports [13]. In addition to treating existing injuries, chiropractic care plays a crucial role in injury prevention, biomechanical optimization, and long-term physical maintenance for athletes. It has been demonstrated that chiropractic interventions, such as spinal manipulation and soft tissue therapies, are effective in the prevention and treatment of sports-related injuries, such as muscle strains and joint sprains [2]. In addition, these techniques contribute to improved biomechanics by enhancing joint mobility, muscle flexibility, and neuromuscular coordination, which ultimately results in improved athletic performance [2,4]. The recovery and maintenance of an athlete's physical health are also significantly influenced by chiropractic care. By addressing musculoskeletal imbalances and promoting proper biomechanics, chiropractors help athletes recover from training sessions and competitions more efficiently, thereby reducing the risk of overuse injuries and facilitating long-term health and performance [14]. Hence, the incorporation of chiropractic care into an athlete's healthcare and training regimen is essential for optimal performance, injury prevention, and physical maintenance.

Evidence-based research on chiropractic care for athletes

Research demonstrating the efficacy of chiropractic care for athletes in addressing sports-related injuries and enhancing performance has highlighted its effectiveness. Numerous studies have reported positive outcomes in chiropractic-treated athletes, including improved musculoskeletal function, reduced pain, and enhanced recovery [1,2]. One study involving judo athletes found that cervical spine manipulation led to a significant increase in grip strength, highlighting the potential of chiropractic care to improve athletic performance [6]. Another study examining the role of sports chiropractors in the National Football League (NFL) demonstrated the benefits of chiropractic care in injury management and overall player health [4]. There are still gaps in research despite the growing body of evidence supporting the incorporation of chiropractic care into sports medicine. Hence, further research is required to examine the long-term effects of chiropractic interventions on athlete performance and the potential benefits of combining chiropractic care with other healthcare modalities for optimal outcomes. In addition, more research is necessary to determine the most effective treatment protocols for specific sports and injury types, which will ultimately contribute to the development of evidence-based guidelines for chiropractic care in sports medicine.

The sports industry in China and Hong Kong

In recent years, the sports industry in China and Hong Kong has experienced significant growth, driven by increased government investment, a growing middle class, and a greater focus on health and wellness. The market size of China's sports industry alone reached approximately 3.12 trillion yuan in 2021 and is projected to continue its rapid growth [15]. Key sectors in this industry include sports infrastructure, professional leagues, sporting goods manufacturing, and sports-related services such as coaching, sports medicine, and fitness facilities [16].

The sports industry in Hong Kong is also thriving, with an increase in demand for fitness and wellness services, as well as the hosting of high-profile international events. For example, the Chief Executive’s 2022 policy also addresses developing sports as an industry and raising the level of professionalism in the sports sector. This includes setting up training and competition grounds for qualified sports clubs and aiding local sports clubs in their participation in significant regional and mainland sporting events [17]. The governments of both China and Hong Kong have enacted policies and initiatives to support the growth of the sports industry. For instance, China's National Fitness Program aims to increase the participation of the population in physical activity and foster the growth of sports-related businesses [18]. Similarly, the government of Hong Kong has implemented strategies for enhancing sports infrastructure, cultivating local athletes, and attracting international events [8]. The continued expansion of the sports industry in China and Hong Kong presents numerous opportunities for enhancing sports medicine services, such as the incorporation of chiropractic care, to promote athlete performance and population health.

The current state of chiropractic care in China and Hong Kong

The current state of chiropractic care in China and Hong Kong shows an optimistic picture, with the availability of chiropractic services increasing. The Sports Chiropractic Council of Hong Kong, China (SCCHK) was founded by the Chiropractic Doctors Association of Hong Kong (CDAHK), as a national representative organization of the International Federation of Sports Chiropractic (FICS) [8]. The CDAHK has recruited chiropractors to work closely with sports groups to raise the value of sports chiropractic in sports medicine. Sports chiropractors mostly hold advanced postgraduate diplomas or degrees from universities, the Fédération Internationale de Football Association (FIFA), or FICS [19]. The SCCHK ensures that athletes in Hong Kong and China have access to chiropractic care as part of the sports healthcare team [8]. 

In Hong Kong, numerous sports associations and teams, such as the Wrestling Association of Hong Kong China, Baseball Association of Hong Kong China, Hoiking Sports Association of Football Association of Hong Kong China, Hong Kong Dodgeball Association, and Mixed Martial Arts Association of Hong Kong China, have chiropractors dedicated to their athletes [8]. In mainland China, sports chiropractors have also been active in serving many Chinese National Teams and major competitions (Table 1) [8]. The public's understanding and acceptance of chiropractic services as a vital component of sports medicine and overall health continues to evolve, but additional efforts are required to promote the public's perception and acceptance of chiropractic services as an integral component of sports medicine and overall health. Hong Kong chiropractors have been treating musculoskeletal diseases [20-39]. Increased collaboration between chiropractors, other healthcare professionals, and sports organizations can help raise the profile of chiropractic care and highlight its importance in promoting athletic performance and health.

**Table 1:** Chiropractic care at the professional sports teams of Hong Kong and China In Hong Kong, Drs. William MH Wan, Alvin Or, Shun Zhe Piong, Eric CP Chu, and Wesly Ku offer dedicated chiropractic services to numerous sports teams and their athletes [8]. In mainland China, Li Peng and teams from the Global Initiative China of Life University have also been active in serving the Chinese National Teams [8]

	Professional sports teams that employ chiropractors	Sports leagues and events with chiropractors' participation
Hong Kong	Wrestling Association of Hong Kong China	Hong Kong Premier League
	Baseball Association of Hong Kong China	2022 World Dodgeball Championship
	Hoiking Sports Association of Football Association of Hong Kong China	
	Hong Kong Dodgeball Association	
	Mixed Martial Arts Association of Hong Kong China	
China	Chinese National Women’s Volleyball Team	2022 Beijing Winter Olympic Games
	Chinese National Swimming Team	2020 Tokyo Olympic Games
	Chinese National T&F Team	2018 Asian Games Jakarta
	Chinese National Badminton Team	2019 ICF Canoe Sprint World Championships Szeged, Hungary
	Chinese National Table Tennis Team	2019 World Rowing Championships Austria
	Chinese National Trampoline Team	2018 and 2019 World Rowing Cups
	Chinese National Cycling Team	2019 ICF Canoe Sprint World Cup
	Chinese National Rowing Team	2020 Chinese National Swimming Competition
	Chinese National Canoe & Kayak Team	
	Chinese National Weightlifting Team	
	Chinese National Women’s Basketball Team	
	Chinese National Synchronizing Swimming Team	
	Chinese National Diving Team	
	Chinese National Archery Team	
	Chinese National Gymnastics Team	
	Chinese National Speed Skating Team	
	Chinese National Figure Skating Team	
	Chinese National Curling Team	

The potential impact of chiropractic care on the sports industry

The integration of chiropractic care into the sports industry has the potential to have a significant impact on various aspects of athletic performance, health, and competitiveness. By addressing biomechanical imbalances and optimizing neuromuscular function, chiropractic care has the potential to boost athlete performance, resulting in increased competitiveness on local, national, and international levels [13]. Moreover, the preventive and rehabilitative nature of chiropractic interventions can contribute to decreased injury rates and improved long-term health among athletes, thereby promoting career longevity and a higher quality of life after retirement [11].

Positive outcomes of chiropractic care in sports medicine can also act as a magnet for international events and investments in the sports industry. By demonstrating the efficacy of chiropractic care in enhancing athlete performance and injury management, countries such as China and Hong Kong can demonstrate their dedication to sports excellence and innovation, thereby attracting major sporting events, global partnerships, and foreign investment. This in turn can result in increased economic opportunities, the creation of new jobs, and the expansion of the sports industry as a whole. Therefore, the incorporation of chiropractic care into sports medicine not only benefits individual athletes but also contributes to the growth and success of the entire sports industry.

Integrating chiropractic care into the sports industry 

**Figure 1:**
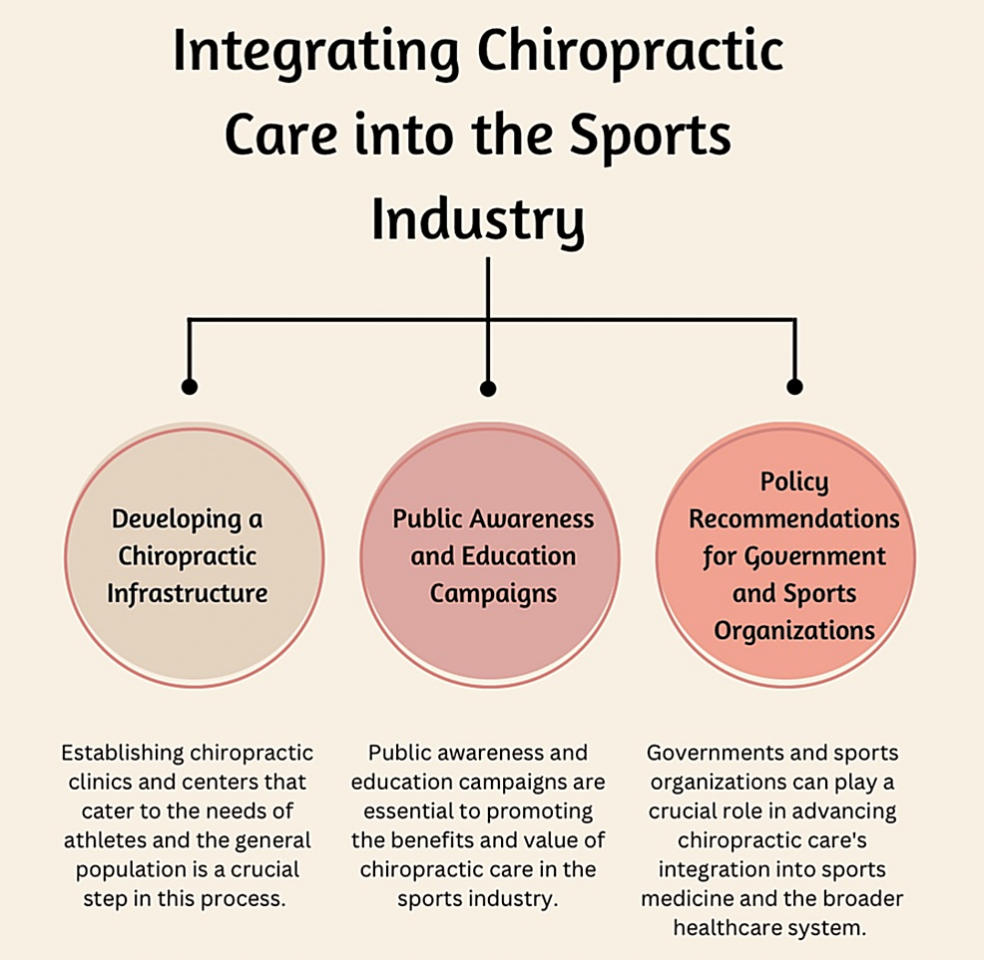
Integrating chiropractic care into the sports industry Integrating chiropractic care into the sports industry requires a multi-pronged approach, including the development of a chiropractic care infrastructure, public awareness and educational campaigns, and supportive policies

Integrating chiropractic care into the sports industry requires a multi-pronged approach, including the development of chiropractic care infrastructure, public awareness and educational campaigns, and supportive policies (Figure 1).

Developing a Chiropractic Care Infrastructure

Developing a robust infrastructure for chiropractic care is essential for promoting the expansion and incorporation of chiropractic services within sports medicine and the broader healthcare system. Establishing chiropractic clinics and centers that cater to the needs of athletes and the general population is a crucial step in this process. These facilities should be outfitted with cutting-edge diagnostic and treatment equipment, thereby enabling chiropractors to provide evidence-based, high-quality care [40]. 

Concurrently, expanding chiropractic education and training programs is essential for generating a skilled labor force capable of meeting the rising demand for chiropractic services. This can be accomplished through the establishment of new chiropractic schools or the incorporation of chiropractic courses into existing sports medicine and healthcare curricula. In addition, offering continuing education opportunities to practicing chiropractors will ensure that they are abreast of the most recent scientific and clinical developments [19].

Collaboration and networking are essential for the development of a robust infrastructure for chiropractic care. The formation of national and regional chiropractic associations that bring together practitioners, educators, researchers, and other stakeholders can facilitate this. Fostering interdisciplinary collaboration between chiropractors and other healthcare professionals, such as physiotherapists, sports medicine physicians, and athletic trainers, will promote a more comprehensive and integrated approach to athlete care and injury management [4]. Ultimately, these efforts will contribute to the widespread acceptance of chiropractic care as an integral part of sports medicine and public health.

Public Awareness and Educational Campaigns

Public awareness and education campaigns are essential to promoting the benefits and value of chiropractic care in the sports industry. Primary audiences for these campaigns should be athletes, coaches, and sports organizations, as they are directly involved in sports performance and decision-making. By using social media and public events, such as seminars, workshops, and sporting events, it is possible to effectively disseminate information and engage with these target groups [41].

Collaboration with local sports celebrities and influencers can greatly increase the reach and effectiveness of chiropractic awareness campaigns. By sharing their personal experiences and testimonials regarding the benefits of chiropractic care, these public figures can influence the perception and acceptance of chiropractic services among the athletic community and the general public [42]. In addition, partnering with sports organizations, educational institutions, and other healthcare professionals can help create a unified front to advocate for the incorporation of chiropractic care into sports medicine and public health initiatives. 

Educational campaigns should emphasize the benefits of chiropractic care that are supported by evidence, such as enhanced performance, injury prevention, and rehabilitation support. Athletes, coaches, and sports organizations will be able to make informed decisions regarding the incorporation of chiropractic care into their training and healthcare strategies if they are provided with accurate, easily accessible, and scientifically based information. Ultimately, public awareness and education campaigns that are well-executed can significantly contribute to the growth and recognition of chiropractic care as an essential component of sports medicine.

Policy Recommendations for Government and Sports Organizations

Governments and sports organizations can play a crucial role in advancing chiropractic care's integration into sports medicine and the broader healthcare system. First and foremost, they should consider incorporating chiropractic care into sports medicine policies and guidelines, given its potential to enhance athletic performance and decrease injury rates. Governments and sports organizations can foster chiropractic care's expansion and acceptance by recognizing it as a valuable component of athlete healthcare. 

Financial incentives should be made available to promote the growth and accessibility of chiropractic services and education. This can include funding for chiropractic clinics, scholarships for chiropractic degree-seeking students, and grants for continuing education and research initiatives. Governments and sports organizations can contribute to the development of a skilled workforce capable of meeting the growing demand for chiropractic care in the sports industry by investing in the chiropractic profession [43]. 

Collaboration with international chiropractic organizations, such as the World Federation of Chiropractic and FICS, can provide invaluable resources and guidance for the development of infrastructure and educational programs for chiropractic care. Establishing partnerships and exchange programs with these organizations can facilitate the exchange of best practices, research, and innovations in chiropractic care, thereby advancing the profession as a whole [44]. Governments and sports organizations can ultimately contribute to improving the health and performance of athletes and the general population by implementing these policy recommendations.

## Conclusions

Chiropractic care has the potential to significantly improve the performance, health, and well-being of athletes, making it an essential component of sports medicine. By addressing biomechanical imbalances and optimizing neuromuscular function, chiropractic care can improve athletes' competitiveness, reduce injury rates, and provide long-term health benefits. For nations such as China and Hong Kong, adopting chiropractic care in the sports industry can result in help attracting international events and investments, thereby fostering economic growth and innovation. Developing a robust infrastructure for chiropractic care, raising public awareness, and implementing supportive policies are essential for realizing these benefits. Governments, sports organizations, healthcare professionals, and athletes must collaborate to promote and integrate chiropractic care, thereby contributing to a healthier, more competitive, and thriving sports community.
